# Comparison of Diagnostic and Therapeutic Protocols for Chronic Obstructive Pulmonary Disease (COPD): Global Initiative for Chronic Obstructive Lung Disease (GOLD) vs. American Thoracic Society/European Respiratory Society (ATS/ERS) Guidelines

**DOI:** 10.7759/cureus.102479

**Published:** 2026-01-28

**Authors:** Nino Basoshvili

**Affiliations:** 1 Cardiology, 5th Clinical Hospital Open Heart, Tbilisi, GEO

**Keywords:** ats/ers guidelines, blood eosinophils, copd: chronic obstructive pulmonary disease, gold 2025 guidelines, inhaled corticosteroids, lower limit of normal (lln), phenotypes, precision medicine, pulmonology, spirometry

## Abstract

Chronic obstructive pulmonary disease (COPD) is a leading cause of global morbidity and mortality. International clinical practice guidelines, specifically the 2025 Global Initiative for Chronic Obstructive Lung Disease (GOLD) and the joint American Thoracic Society/European Respiratory Society (ATS/ERS) guidelines, provide standardized frameworks for its diagnosis and management. This narrative review compares these two frameworks, highlighting the trade-off between GOLD’s clinical simplicity and the ATS/ERS emphasis on physiological precision. Key areas of divergence include diagnostic thresholds (fixed ratio vs. lower limit of normal), treatment classification systems (GOLD’s ABE tool vs. ATS/ERS phenotype-driven care), and the use of biomarkers like blood eosinophils. Understanding these nuances is critical for clinicians to optimize diagnostic labeling and long-term therapeutic outcomes.

## Introduction and background

Millions of people worldwide suffer from chronic obstructive pulmonary disease (COPD), a progressive inflammatory condition that imposes a significant clinical and economic burden [1]. Reducing exacerbations-sudden episodes of worsening respiratory symptoms-and enhancing patient quality of life are the primary goals of management [2]. To standardize care, clinicians rely on recommendations from the Global Initiative for Chronic Obstructive Lung Disease (GOLD) and the joint American Thoracic Society (ATS) and European Respiratory Society (ERS) guidelines [1-6].

GOLD provides a pragmatic strategy designed for broad clinical applicability, particularly in primary care [1]. In contrast, the joint ATS/ERS frameworks emphasize methodological rigor and individualized care based on physiological precision [6].

A central point of divergence is the interpretation of spirometry, the gold-standard test used to measure lung function [1,6]. Guidelines differ on how to use the FEV1/FVC ratio, which measures the volume of air a person can forcefully exhale in one second (FEV1) compared to the total volume they can exhale (FVC). GOLD utilizes a fixed ratio (<0.70), which is easy to apply but may lead to overdiagnosis in the elderly [1-3]. ATS/ERS recommends using the lower limit of normal (LLN) and Z-scores, which are statistical methods that compare a patient’s results to healthy individuals of the same age, sex, and height to ensure diagnostic accuracy [6].

## Review

Methods

To ensure replicability and transparency, this manuscript follows a structured narrative review methodology.

Search Strategy

A comprehensive search was conducted across PubMed/MEDLINE, Google Scholar, and the official repositories of GOLD, ATS, and ERS for documents published or updated between January 2017 and January 2025 [1-6].

Keywords

Keywords included "GOLD 2025," "ATS/ERS COPD," "spirometry interpretation," "eosinophils," and "triple therapy."

Inclusion Criteria

Documents were included if they were primary guideline reports (e.g., GOLD 2025 Report), technical standards for spirometry interpretation, or pivotal randomized controlled trials (RCTs) that directly informed these guidelines [1-6].

Synthesis

This review provides a qualitative comparison of diagnostic and therapeutic recommendations. It is a narrative review and does not include a meta-analysis or new quantitative synthesis of raw trial data.

Diagnosis: simplicity vs. precision

The diagnosis of COPD requires post-bronchodilator spirometry to confirm persistent airflow limitation [1-6].

GOLD Approach: Fixed Ratio

GOLD defines airflow obstruction as a post-bronchodilator FEV_1_/FVC < 0.70 [1].

Severity staging: GOLD categorizes airflow limitation into four stages based on FEV_1_, the percent predicted [1]; that is, (i) GOLD 1 (mild): predicted; (ii) GOLD 2 (moderate): predicted; (iii) GOLD 3 (severe): predicted; and (iv) GOLD 4 (very severe): predicted.

Rationale: This fixed threshold prioritizes ease of use and broad applicability in routine clinical practice. However, it carries a risk of overdiagnosis in older adults and underdiagnosis in younger populations as lung function naturally changes with age [3].

ATS/ERS Approach: LLN and Z-Scores

ATS/ERS guidelines advocate for defining obstruction using the lower limit of normal (LLN), derived from population-based reference equations and expressed as Z-scores [6].

Precision: By accounting for age, sex, height, and ethnicity, this method reduces systematic diagnostic bias and allows for a more nuanced interpretation of early or borderline airflow limitation [3-6].

Quality: This framework places a stronger emphasis on spirometry quality control and standardized testing techniques [6].

Treatment: algorithm vs. individualization

Both frameworks share core principles: symptom assessment using scores like the mMRC (modified Medical Research Council) or CAT (COPD Assessment Test), exacerbation risk stratification, and individualized pharmacologic therapy [1-6].

GOLD: ABE Assessment Tool

GOLD utilizes a structured, algorithm-driven approach to guide initial therapy [1]. The recent 2023-2025 updates replaced the traditional ABCD system with the ABE grouping.

Group A (low symptoms, low risk): mMRC 0-1 or CAT; 0-1 moderate exacerbations (Initial treatment: any bronchodilator) [1].

Group B (high symptoms, low risk): mMRC or CAT ; 0-1 moderate exacerbations (Initial treatment: long-acting bronchodilator (LABA or LAMA); LABA + LAMA preferred if symptoms are significant) [1].

Group E (exacerbation-prone): moderate exacerbations or hospitalization (Initial treatment: dual bronchodilation (LABA + LAMA)) [1].

Biomarkers and Inhaled Corticosteroids (ICS)

GOLD uses** **blood eosinophil counts** **as a biomarker to guide the addition of inhaled corticosteroids (ICS) to bronchodilator therapy [1-2].

High benefit (cells/µL): strong recommendation for ICS use to prevent frequent exacerbations.

Low benefit (cells/µL): ICS use is discouraged due to limited efficacy and an increased risk of pneumonia [1-2].

ATS/ERS: Phenotype-Driven Care

In contrast, ATS/ERS recommendations place greater emphasis on individualized clinical judgment** **rather than rigid algorithms [6].

Therapeutic stance**: **These guidelines adopt a more cautious stance regarding ICS, emphasizing a risk-benefit balance to avoid adverse effects like pneumonia [6].

De-escalation: ATS/ERS strongly encourage periodic reassessment and the de-escalation (withdrawal) of ICS if patients do not show clear benefit or develop complications [6].

Emerging Therapies and Future Directions

While not yet standard in current guidelines, several novel therapies show promise in ongoing research.

Dual PDE3/4 inhibitors: Ensifentrine offers both bronchodilatory and non-steroidal anti-inflammatory effects [4].

Targeted biologics: Clinical trials, such as the phase 3 Pivotal Study to Assess the Efficacy, Safety and Tolerability of Dupilumab in Patients With Moderate-to-severe COPD With Type 2 Inflammation (BOREAS), have demonstrated the efficacy of dupilumab (targeting IL-4/IL-13) in reducing exacerbations for patients with type 2 inflammation [5]. Mepolizumab also targets eosinophilic COPD phenotypes.

Early detection: Advanced imaging and digital biomarkers are being researched to identify "early COPD" phenotypes before traditional spirometric thresholds are crossed [3].

Discussion

The comparison of GOLD and ATS/ERS recommendations reveals two complementary paradigms [1-6] (Table 1). GOLD offers clinical simplicity through its fixed diagnostic ratio and algorithmic ABE treatment grouping, facilitating ease of application in primary care. In contrast, the ATS/ERS framework prioritizes accuracy through the use of LLN and Z-score interpretation [1-6], which reduces diagnostic misclassification in an aging population.

**Table 1:** Comparison of diagnostic protocols GOLD: Global Initiative for Chronic Obstructive Lung Disease; ATS/ERS: American Thoracic Society/European Respiratory Society; FEV1: forced expiratory volume in 1 second; FVC: forced vital capacity

Aspect	GOLD guidelines	ATS/ERS guidelines
Diagnostic spirometry	Post-bronchodilator FEV1​/FVC<0.70	Post-bronchodilator FEV1​/FVC below lower limit of normal (LLN)
Rationale	Simple, easy to apply in clinical practice	Reduces age- and sex-related misclassification
Use of reference values	Percent predicted values	Z-scores based on population reference equations
Early/borderline disease	Less emphasis on borderline obstruction	Detailed interpretation of early airflow limitation
Spirometry quality	Recommended	Strongly emphasized with standardized techniques
Risk of misclassification	Possible overdiagnosis in the elderly	More precise, but less practical in low-resource settings

Therapeutically, GOLD provides clear thresholds for biomarker-guided escalation (Table 2). Conversely, ATS/ERS advocates for a more safety-focused, response-based strategy with a strong emphasis on continuous reassessment and the de-escalation of therapy when risks outweigh benefits [6]. As precision medicine evolves, future guideline updates are likely to move toward more integrated, phenotype-driven strategies.

**Table 2:** Comparison of therapeutic strategies GOLD: Global Initiative for Chronic Obstructive Lung Disease; ATS/ERS: American Thoracic Society/European Respiratory Society; LABA: long-acting beta-agonist; LAMA: long-acting muscarinic antagonist; COPD: chronic obstructive pulmonary disease

Treatment aspect	GOLD	ATS/ERS
Initial treatment	Based on symptom burden and exacerbation risk (ABE grouping)	Individualized based on symptoms, lung function, and exacerbation history
Bronchodilators	LABA or LAMA as first-line	LABA or LAMA; emphasizes patient response
Dual bronchodilation	LABA + LAMA for persistent symptoms	Strongly supported for moderate to severe COPD
Inhaled corticosteroids (ICS)	Recommended in patients with frequent exacerbations and blood eosinophils ≥300 cells/µL	More cautious use; emphasizes risk–benefit assessment
Triple therapy	LABA + LAMA + ICS for severe disease	Supported in selected high-risk patients
Non-pharmacologic care	Pulmonary rehabilitation, smoking cessation, vaccination	Strong emphasis on pulmonary rehabilitation and education

## Conclusions

GOLD and ATS/ERS remain essential resources for the diagnosis and management of COPD. GOLD prioritizes simplicity and broad applicability, whereas ATS/ERS emphasizes diagnostic precision and individualized interpretation. To maximize patient outcomes, clinicians should adhere to established guideline-directed care while remaining informed about emerging biologic and digital phenotyping strategies that may soon redefine the standard of care.
